# Reproducibility and discriminant validity of two clinically feasible measurement methods to obtain coronal plane gait kinematics in participants with a lower extremity amputation

**DOI:** 10.1371/journal.pone.0217046

**Published:** 2019-05-21

**Authors:** Ruud A. Leijendekkers, Thomas J. Hoogeboom, Gerben van Hinte, Lars Didden, Thomas Anijs, Maria W. G. Nijhuis-van der Sanden, Nico Verdonschot

**Affiliations:** 1 Department of Orthopaedics, Physical Therapy, Radboud University Medical Centre, Nijmegen, the Netherlands; 2 Radboud Institute for Health Sciences, IQ Healthcare, Radboud University Medical Centre, Nijmegen, the Netherlands; 3 Radboud Institute for Health Sciences, Orthopaedic Research Laboratory, Radboud University Medical Centre, Nijmegen, the Netherlands; 4 Department of Rehabilitation, Radboud University Medical Centre, Nijmegen, the Netherlands; 5 Laboratory for Biomechanical Engineering, University of Twente, Enschede, the Netherlands; Holland Bloorview Kids Rehabilitation Hospital, CANADA

## Abstract

**Introduction:**

Measuring coronal plane gait kinematics of the pelvis and trunk during rehabilitation of participants with a lower extremity amputation is important to detect asymmetries in gait which are hypothesised as associated with secondary complaints. The aim of this study was to test the reproducibility and discriminant validity of a three-dimensional (3-D; inertial measurement units) and a two-dimensional (2-D; video-based) system.

**Methods:**

We tested the test-retest and inter-rater reproducibility of both systems and the 2-D system, respectively, in participants with a lower extremity amputation (group 1) and healthy subjects (group 2). The discriminant validity was determined with a within-group comparison for the 3-D system and with a between-group comparison for both systems.

**Results:**

Both system showed to be test-retest reliable, both in group 1 (2-D system: ICC3.1_agreement_ 0.52–0.83; 3-D system: ICC3.1_agreement_ 0.81–0.95) and in group 2 (3-D system: ICC3.1_agreement_ 0.33–0.92; 2-D system: ICC3.1_agreement_ 0.54–0.95). The 2-D system was also inter-rater reliable (group 1: ICC2.1_agreement_ 0.80–0.92; group 2: ICC2.1_agreement_ 0.39–0.90). The within-group comparison of the 3-D system revealed a statistically significant asymmetry of 0.4°-0.5° in group 1 and no statistically significant asymmetry in group 2. The between-group comparison revealed that the maximum amplitude towards the residual limb (MARL) in the low back (3-D system) and the (residual) limb—trunk angle (2-D system) were significantly larger with a mean difference of 1.2° and 6.4°, respectively, than the maximum amplitude of healthy subjects. However, these average differences were smaller than the smallest detectable change (SDC) of group 1 for both the MARL (SDC_agreement_: 1.5°) and the residual limb—trunk angle (SDC_agreement_: 6.7°-7.6°).

**Conclusion:**

The 3-D and 2-D systems tested in this study were not sensitive enough to detect real differences within and between participants with a lower extremity amputation and healthy subjects although promising reproducibility parameters for some of the outcome measures.

## Introduction

Asymmetries in spatiotemporal [1–6], kinematic [7–12] and kinetic [9,12,13] parameters during gait are common in individuals with a lower extremity amputation. These asymmetries are associated with increased metabolic energy cost [14–16] and low back pain [17,18]. Especially, the asymmetries in the coronal plane kinematic parameters of the pelvis and trunk lead to increased joint and muscle forces and may therefore contribute to higher low back injury risk [18–20]. Low back pain is in 52–84% of the individuals with a lower extremity amputation a secondary disability [17,18,21–23]. Therefore, rehabilitation programmes typically focus on optimising gait symmetry [14,24–26].

Evaluating gait parameters in daily clinical practice to track the patient’s progress during the rehabilitation is challenging. Spatiotemporal parameters can be measured quite easily with a transducer [27] or ‘GAITRite walkway system’ [28,29]. To obtain kinematic and kinetic parameters, the use of a three-dimensional (3-D) motion capture system such as ‘Vicon’ is a widely used and accepted method [30–32]. There is some debate about the reproducibility [33], but most importantly it is not suitable in daily clinical practice because it is high in cost, time-consuming and not portable [31,34]. The ‘gait real-time analysis interactive lab’ (GRAIL) [35] and ‘computer-assisted research environment’ (CAREN) [14] have an added option to use real-time visual feedback and virtual reality to enhance the integration of these systems in rehabilitation programmes. However, both systems have the same limitations as the Vicon system.

Alternatively, wireless movement analysis systems using body-worn sensors (e.g. ValedoMotion from Hocoma or MVN Biomech from Xsens) can be used: they are cheaper and easy to use. Additionally, they have the ability to provide real-time visual feedback [36–40]. These clinical feasible systems measure the 3-D angular tilt and velocity of body segments with respect to magnetic fields and gravity using multiple small light weight inertial measurement units (IMU) [41,42]. The degree of accuracy and reproducibility is specific to the used anatomical landmark and IMU-system [37,43]. Another option for obtaining kinematic data is using a two-dimensional (2-D) video-based system (e.g. Dartfish from Dartfish Inc.) [44,45], which is also inexpensive and easy to use, although current drawbacks are the absence of real-time visual feedback and the limited validity compared to 3-D systems because the measured kinematic constructs differ from each other [44–46].

**Fig 1:**
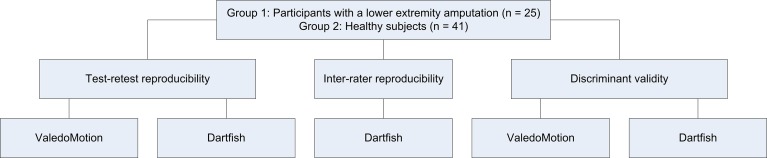
Flowchart study. ValedoMotion: the inertial measurement units system; Dartfish: the two-dimensional video-based system.

The aim of this cross-sectional study was to test the reproducibility and discriminant validity (whether a method was able to detect asymmetries and/or distinguish groups) of two measurement methods for collecting coronal plane gait kinematics in participants with a lower extremity amputation in daily clinical practice. We obtained the kinematic parameters using both an IMU-system and a 2-D video-based system. Additionally, we measured healthy subjects with both systems in order to gather norm values and to determine the discriminant validity (Fig 1).

## Methods

We tested the test-retest reproducibility and the discriminant validity of both an IMU-system (ValedoMotion, Hocoma, Volketswil, Switzerland) and a 2-D video-based system (Dartfish-software, Dartfish, Fribourg, Switzerland). We also determined the inter-rater reproducibility of the 2-D video-based system. A priori we verified the accuracy of the IMU-system using a goniometer in an experimental setting (S1 Text. Appendix A). This experiment showed that the IMU system had an excellent accuracy, but a slight asymmetry (0.2 to 0.7 degree) in maximum amplitude between left and right was present. The COSMIN Checklist was followed for the preparation of the manuscript to ensure methodological quality [47].

### Participants

Two groups participated in this study (Fig 1). Group 1: all consecutive individuals with a lower extremity amputation who completed a rehabilitation program in our centre or had a regular follow-up within a time period of three months were eligible for the study if they walked unaided. Group 2: healthy subjects who were relatives or acquaintances of the researchers were approached to participate in the study. Prior to the assessment a written informed consent was obtained from all participants. The study was conducted according to the principles of the Declaration of Helsinki (64th version, 19-10-2013). The protocol (registration number 2012/547) was approved by the Ethics Committee of the Radboud university medical centre. The individuals in this manuscript have given written informed consent (as outlined in PLOS consent form) to publish these case details.

### Study procedure

The test-retest assessment was performed by a physiotherapy student (BvD), following training from an experienced physiotherapist (RL). The training included a pilot measurement in three healthy individuals to ensure standardisation of the procedure. Both, the test- and retest-assessment were performed consecutively in one session on the same day. The participants were offered sufficient time (at least 5 minutes) to rest between the two assessments. During an assessment the participant wore simultaneously two IMU’s and four tape-markers. The tape-markers were used to facilitate the analysis with the 2-D video-based system. The IMU’s and tape-markers were removed after the first assessment and reattached after the break.

The discriminant validity of both the IMU system and the 2-D video-based system were determined using the data of the test-assessment.

### Testing procedure

The IMU-system consisted of three wireless IMU’s containing a tri-axillar gyroscope, magnetometer, and accelerometer with a fixed axle system. Two of the IMU’s were designed for positioning on an anatomical landmarks (low back and pelvis) using double-sided tape, the third IMU was designed for calibrating the low back and pelvic IMU using an additional reference holder including a level instrument (S1 Text. Appendix A, Fig A.3). In the calibration process the orientations of the IMU’s following the magnetometer are used to determine the zero-angles, which are the measured rotation angles at which the patient is standing upright. The calibrated rotations were measured using a sampling frequency of 300 Hz and exported to a notebook (Hewlett-Packard).

**Fig 2:**
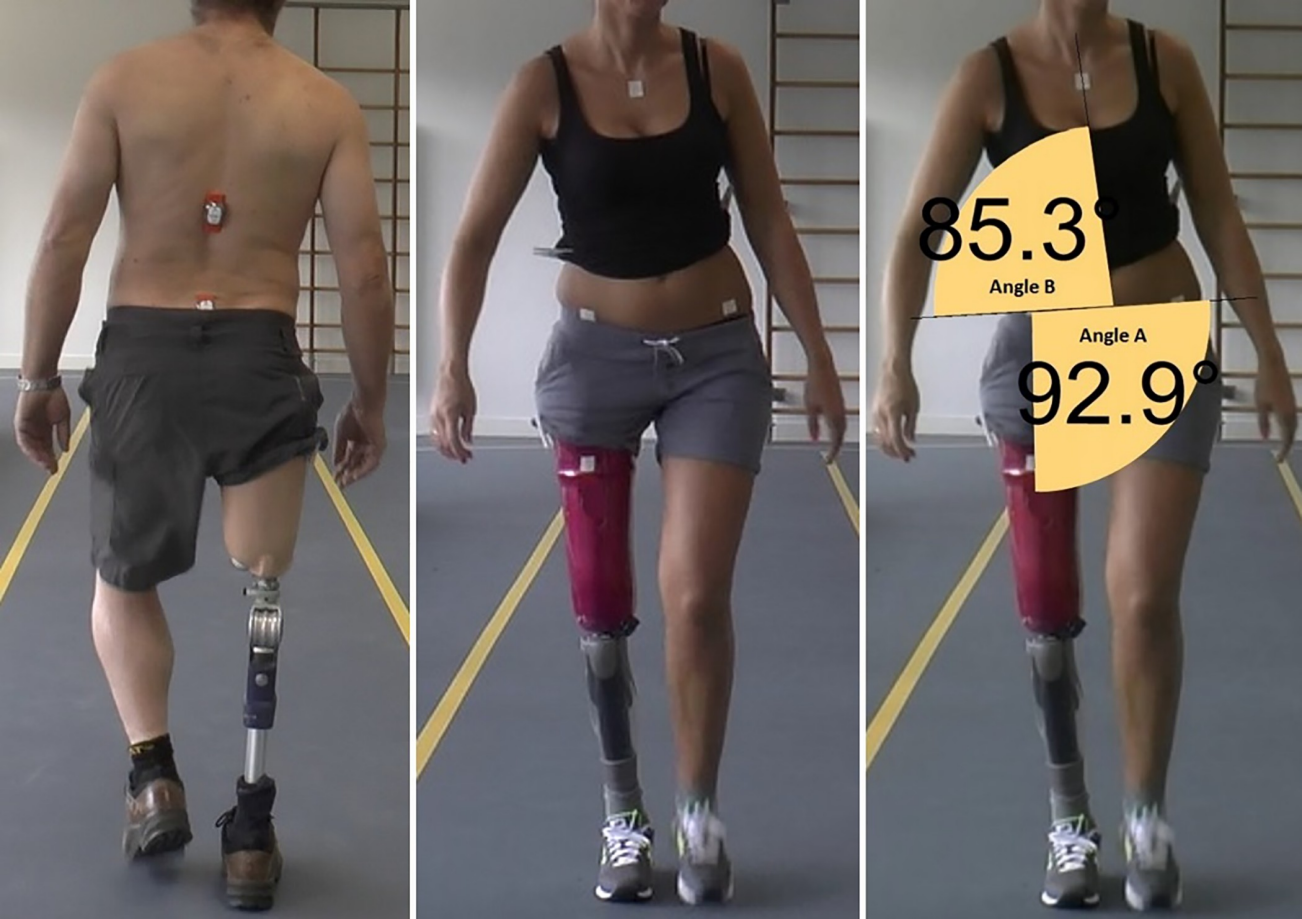
Position of the IMU’s and tape-markers. Left: low back (upper) and pelvic (lower) IMU; Middle: tape-markers; Right: on-screen measurement of angle A = pelvis–limb angle and angle B = pelvis—trunk angle using Dartfish-software. Limb—trunk angle = *A + (180—B)* = 187,6° (in this example).

Each assessment started with the attachment of IMU’s using double-sided tape. The pelvic IMU was positioned on the basis of the sacrum (at the height of the posterior superior iliac spine). An applicator was used to determine the position of the low back IMU, which was 17.5 centimetre (cm) cranial of the pelvic IMU (Fig 2). Thereafter, the 1.0 by 1.0 cm tape-markers were placed on: 1) the anterior superior iliac spine (ASIS) on both sides, 2) the proximal part of the manubrium, and 3) 30 cm distal of the ASIS on the ventral side of the residual limb or a randomly selected limb in group 1 or group 2, respectively (Fig 2). After calibrating the IMU’s the participants were instructed to walk three times up and down a 15-meter long walkway, on self-selected comfortable walking speed. The gait of the participants was recorded at 50 frames per second (1080/50p) using a camcorder (Panasonic HC-X920, Panasonic Netherlands, 's-Hertogenbosch, The Netherlands). At the start of each assessment day the location of the experiment was checked for magnetic fields using the ValedoMotion software. Participants and researchers where instructed to keep cell phones out of the measuring location to avoid magnetic interference.

### Data analysis

We analysed the coronal plane kinematic parameters of the low back and pelvic IMU. Of each assessment we only analysed the data of the three trials towards the camera because this was the orientation in which the IMU’s were calibrated. MATLAB (Release 2015b, The MathWorks Inc., Natick, Massachusetts, United States) was used to process the csv-file containing the raw data (S1 Text. Appendix A, Fig A.4). First, a filter was used to get rid of low frequencies caused by substantial integration drift picked up over the duration of the trial measurement, and high frequencies as a result of soft-tissue, and skin artefacts and high frequency noise [48,49]. The angle output of the IMU’s was filtered using a band-pass filter (bi-directional 2^nd^ order Butterworth filter, low-pass frequency of 5 Hz, high-pass frequency of 0.3 Hz). Second, the transition in the data between the way towards the camera and the way back was defined manually by an engineer (LD) who performed the data analysis of both the test- and the retest-assessment. Third, a MATLAB protocol was used to automatically detect peak angles for each trial based on an expected cadence interval within 0.5 and 2 Hz; due to the nature of the automated detection, negative peak angles (maximum amplitude to the right; MAR) were identified in between two positive peak values (maximum amplitude to the left; MAL) resulting in an unequal number of peaks to the right and to the left. Fourth, the detected peaks were visually checked and manually corrected if necessary before proceeding. Finally, we calculated the average for both the MAL and the MAR for each assessment. Additionally, the extent of asymmetry within each assessment was calculated as the average difference between MAL and MAR, resulting in a symmetry value. For the healthy subject (group 2), the MAL and MAR of the low back IMU represented the maximum lateral flexion angle to the left or to the right, respectively. The MAL and MAR of the pelvic IMU represented a lateral tilt angle to the left or to the right, respectively. For the participants with a lower extremity amputation (group 1) the direction of the movement was related to the side of the amputation; maximum amplitude towards the residual limb (MARL), maximum amplitude towards the sound limb (MASL), symmetry value (difference between MARL and MASL).

The 2-D video-based system (Dartfish-software) was used for photo goniometry. The video was played in slow motion and frame-by-frame, and was paused during the mid-stance phase of the fourth gait cycle of the second trial towards the camera. This moment was chosen to ensure that the gait was constant and to standardise the measurement. Within the still image the graphical goniometer included in the software was used to perform on-screen measurements. We measured the pelvis—limb (A) and pelvis—trunk angles (B) in degrees using the tape-markers (Fig 2). These angles were used to calculate an overall angle (limb—trunk angle; C) by the formula: *C = A + (180—B)*. The limb—trunk angle represents the posture in coronal plane with respect to global space. A larger angle represents a posture with a greater ipsilateral lateral flexion of the trunk and/or a greater abduction of the (residual) limb during the mid-stance phase. To determine the test-retest reproducibility of the 2-D video-based system the test-assessment was analysed twice with 4 weeks in between by a physiotherapy student (ER). A second physiotherapy student (RvE) assessed the test-assessment as well to establish the inter-rater reproducibility.

### Statistical analysis

Participant characteristics including gender, age, time from primary amputation to inclusion, level of amputation, cause of primary amputation, type of prosthesis, and Medicare Functional Classification Level (MFC-level) [50], also known as ‘K-levels’ (0–4) in which ‘K0’ represents a non-ambulatory person and ‘K4’ a high level prosthesis user, were described. The test-retest reproducibility was determined for both the IMU-system (low back and pelvic IMU) and the 2-D video-based system. The inter-rater reproducibility was only determined for the 2-D video-based system because measurement error during the analysis can be easily introduced by the rater due to the manual data analysis. In the IMU-system the analysis is automated, which decreases the risk on measurement error due to the rater. The difference in angles (degrees) within the test-retest and within the inter-rater assessment was calculated. The discriminant validity of the IMU system was determined by a within-group comparison and between-group comparison. In the within-group comparison the MARL was compared to the MASL (group 1) and the MAL was compared to the MAR (group 2). In the between-group comparison the MARL and MASL (group 1) were compared to the average of MAL and MAR (group 2). The discriminant validity of the 2-D video-based system was determined by a between-group comparison. All data were checked for normality and if applicable for equality of variance. The within-group analysis were evaluated with a paired T-test. The between-group analysis were evaluated with an independent T-test or the Welch test. The categorical data were presented as exact numbers and percentages and were calculated for the various levels. For the continuous data, means and standard deviations were calculated.

Test-retest and inter-rater reproducibility were both divided in reliability and agreement parameters [51]. Reliability was tested using the intraclass correlation coefficient (ICC) with 95% confidence intervals (CI). ICC’s were calculated using a two-way mixed effects model (ICC3.1_agreement_) with 95% CI for the test-retest reliability and using a two-way random effects model (ICC2.1_agreement_) for the inter-rater reliability [52,53]. Agreement was assessed by calculating the standard error of measurement (SEM_agreement_) and the smallest detectable change (SDC_agreement_). Both are expressed in the unit of the measurement (degrees). The SEM was calculated as SEM_agreement_ = √σ^2^_error_ = √(σ^2^_o_+ σ^2^_residual_) [52]. The variance due to systematic differences between the observers (σ^2^_o_) and the residual variance (σ^2^_residual_) were obtained from the varcomp analysis [52]. The SEM_agreement_ was used to calculate the SDC_agreement_ = 1.96 * √n * SEM [51]. In this formula ‘n’ refers to the number of measurements, which is two in our study [51]. A Bland-Altman plot including 95% limits of agreement (95% LoA) was constructed to determine if there was bias in measurement error [53,54]. The interpretation of the ICC values was based on guidelines offered by Byrt [55]: 0.01–0.20 poor accuracy, 0.21–0.51 slight accuracy, 0.41–0.60 fair accuracy, 0.61–0.80 good accuracy, 0.81–0.92 very good accuracy, and 0.93–1.00 excellent accuracy. All analyses were performed using IBM SPSS Statistics v22. In all cases, two sided p-values <0.05 were considered to be statistically significant.

## Results

Group 1 consisted of 25 participants with a lower extremity amputation (21 men) and group 2 consisted of 41 healthy subjects (19 men) as presented in Table 1. The mean age was 51 and 29 years in group 1 and 2, respectively. In group 1, 11 participants used socket-suspended prostheses (transfemoral: 4, through knee: 1, transtibial: 6) and 14 participants used bone-anchored prostheses (transfemoral: 13, transtibial: 1). In 5 healthy subjects a technical error occurred during the data collection with the IMU-system which resulted in undetectable peak values due to an inconsistent waveform, hence only 36 participants were included in the analyses of the IMU-system.

**Table 1:** Participant characteristics.

Participant characteristics			Group 1		Group 2	
			n = 25		n = 41	
Male gender, n (%)			21	(84)	19	(46)
Age (yrs), mean (SD)			50.7	(15.0)	29.2	(12.8)
Time from primary amputation to inclusion (yrs), mean (SD)			17.7	(16.4)	NA	
Amputation level, n (%)						
	- Transfemoral amputation		17	(68)	NA	
	- Through knee amputation		1	(4)	NA	
	- Transtibial amputation		7	(28)	NA	
Cause of primary amputation, n (%)						
	- Trauma		19	(76)	NA	
	- Tumor		4	(16)	NA	
	- Vascular		0	(0)	NA	
	- Other		2	(8)	NA	
MFC-level, n (%)						
	- Level 0		0	(0)	NA	
	- Level 1		0	(0)	NA	
	- Level 2		1	(4)	NA	
	- Level 3		19	(76)	NA	
	- Level 4		5	(20)	NA	
Type of prosthesis, n (%)						
	- Socket-suspended prosthesis		11	(44)	NA	
	- Bone-anchored prostheses		14	(56)	NA	

%: percentage; yrs: years; SD: standard deviation; MFC-level: Medicare Functional Classification Level; NA: not applicable; Group 1: participants with a lower extremity amputation; Group 2: healthy subjects

### Test-retest reproducibility

#### IMU-system

**Fig 3:**
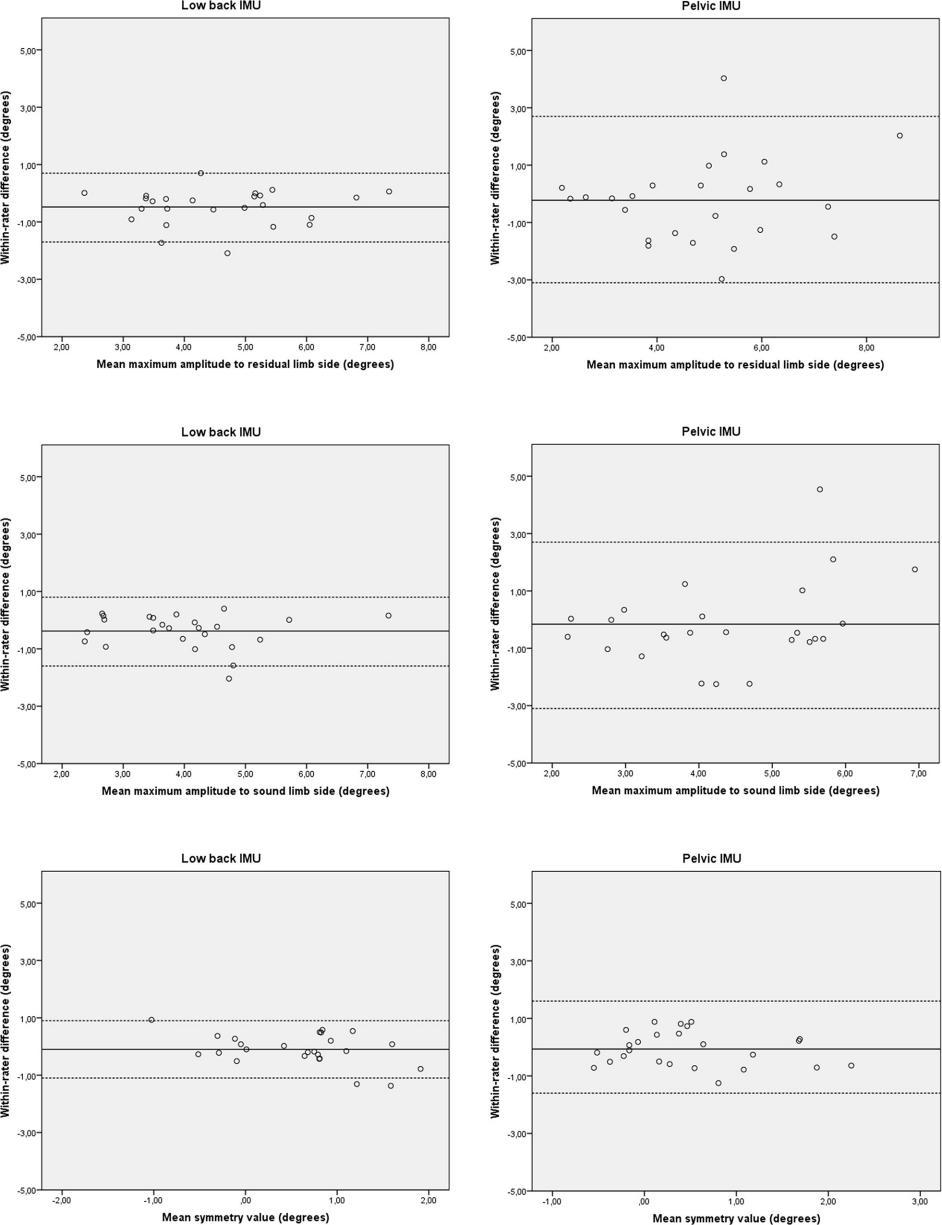
Bland–Altman plots for test-retest reproducibility of the IMU-system in participants with a lower extremity amputation. The solid line represents the mean difference (systematic bias) and the dashed lines illustrate the 95% limits of agreement (mean difference ± 1.96 SD of the difference).

**Fig 4:**
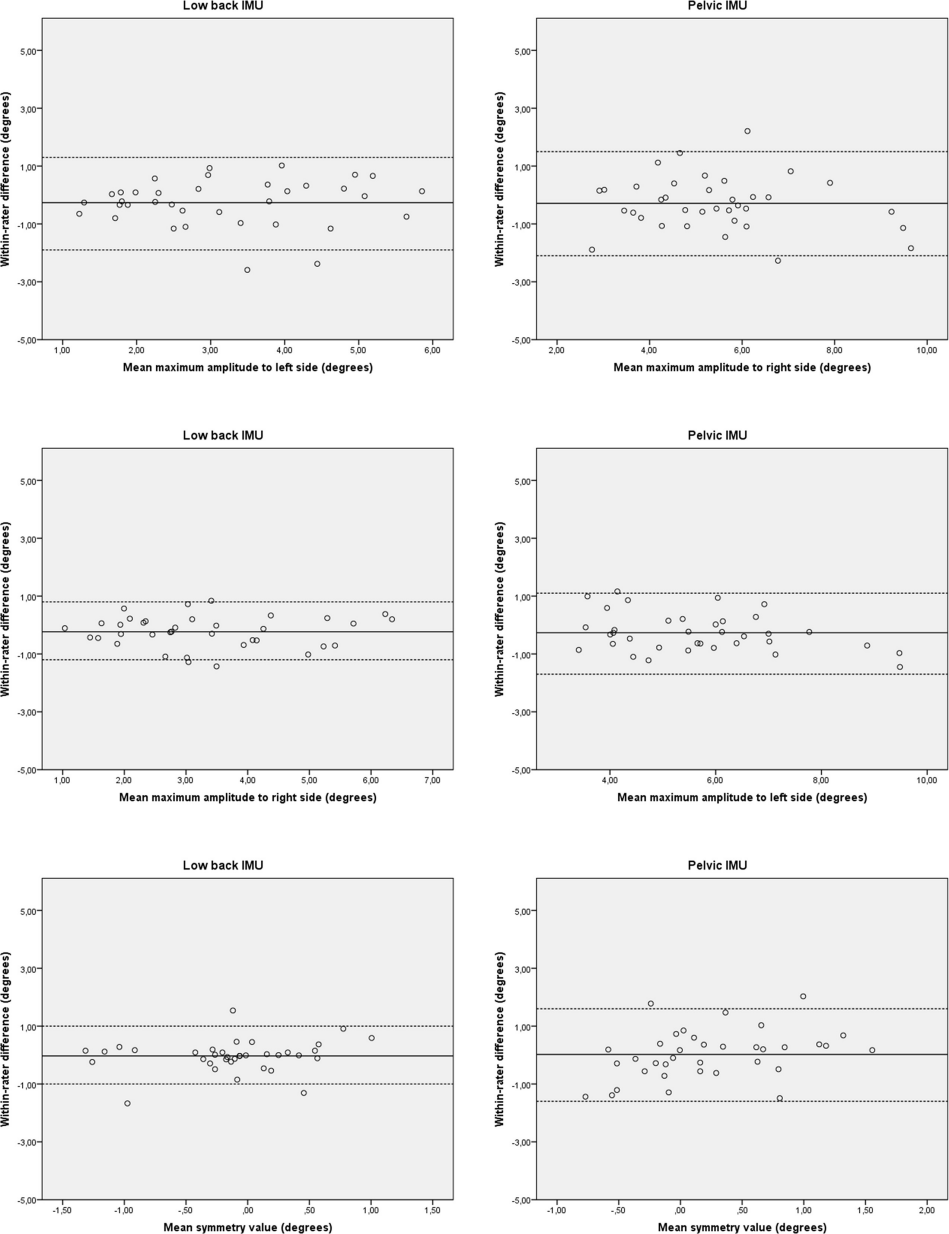
Bland–Altman plots for test-retest reproducibility of the IMU-system in healthy subjects. The solid line represents the mean difference (systematic bias) and the dashed lines illustrate the 95% limits of agreement (mean difference ± 1.96 SD of the difference).

The test-retest reproducibility of the IMU system in group 1 and group 2 is detailed in Table 2. In group 1, the test-retest reliability of the low back IMU and the pelvic IMU was good to very good and fair to good, respectively. The SEM and SDC of the low back IMU ranged from 0.4° to 0.5° and 1.1° to 1.5°, respectively. The SEM and SDC of the pelvic IMU ranged from 1.1° to 1.5° and 1.2° to 2.9°, respectively. In group 2, the test-retest reliability of the low back IMU and the pelvic IMU was good to very good and slight to very good, respectively. The SEM and SDC of the low back IMU ranged from 0.4° to 0.6° and 1.1° to 1.6°, respectively. The SEM and SDC of the pelvic IMU ranged from 0.5° to 0.7° and 1.4° to 1.9°, respectively. In both groups no bias in measurement error was detected as shown in the Bland-Altman plots (Figs 3 and 4).

**Table 2:** Test-retest reproducibility.

IMU / Angle	Test (°)	Retest (°)	Diff test-retest (°)	95% LoA (°)	ICC3.1_agreement_ (95% CI)	SEM_agreement_	SDC _agreement_
	mean (SD)	mean (SD)	mean (SD)			(°)	(°)
**IMU-system**							
***Participants with a lower extremity amputation***							
*Maximum amplitude to residual limb side*: *MARL*							
Low back IMU (n = 25)	4.3 (1.3)	4.8 (1.3)	-0.5 (0.6)	-1.7; 0.7	0.83 (0.45; 0.94)**	0.5	1.5
Pelvic IMU (n = 25)	4.7 (1.9)	5.0 (1.7)	-0.2 (1.5)	-3.1; 2.7	0.66 (0.36; 0.83)**	1.0	2.9
*Maximum amplitude to sound limb side*: *MASL*							
Low back IMU (n = 25)	3.8 (1.2)	4.2 (1.2)	-0.4 (0.6)	-1.6; 0.8	0.84 (0.56; 0.93)**	0.5	1.4
Pelvic IMU (n = 25)	4.3 (1.7)	4.5 (1.3)	-0.2 (1.5)	-3.1; 2.7	0.52 (0.16; 0.76)*	1.0	2.8
*Symmetry (MARL—MASL)*							
Low back IMU (n = 25)	0.5 (0.7)	0.6 (0.9)	-0.1 (0.5)	-1.1; 0.9	0.75 (0.51; 0.88)**	0.4	1.1
Pelvic IMU (n = 25)	0.4 (0.8)	0.5 (0.9)	-0.1 (0.6)	-1.3; 1.1	0.74 (0.49; 0.88)**	0.4	1.2
***Healthy subjects***							
*Maximum amplitude to left*: *MAL*							
Low back IMU (n = 36)	3.1 (1.4)	3.3 (1.3)	-0.3 (0.8)	-1.9; 1.3	0.82 (0.66; 0.90)**	0.6	1.6
Pelvic IMU (n = 36)	5.5 (1.5)	5.8 (1.7)	-0.3 (0.7)	-1.7; 1.1	0.91 (0.82; 0.96)**	0.5	1.4
*Maximum amplitude to right*: *MAR*							
Low back IMU (n = 36)	3.2 (1.4)	3.4 (1.4)	-0.2 (0.5)	-1.2; 0.8	0.92 (0.83; 0.96)**	0.4	1.2
Pelvic IMU (n = 36)	5.3 (1.7)	5.6 (1.8)	-0.3 (0.9)	-2.1; 1.5	0.86 (0.73; 0.92)**	0.7	1.9
*Symmetry (MAL—MAR)*							
Low back IMU (n = 36)	-0.1 (0.7)	-0.1 (0.6)	0.0 (0.5)	-1.0; 1.0	0.62 (0.37; 0.79)**	0.4	1.1
Pelvic IMU (n = 36)	0.2 (0.8)	0.2 (0.6)	0.0 (0.8)	-1.6; 1.6	0.33 (0.00; 0.59)*	0.6	1.6
**2-D video-based system**							
***Participants with a lower extremity amputation***							
Pelvis—residual limb (n = 25)	89.3 (6.7)	89.1 (5.9)	0.2 (2.1)	-3.9; 4.3	0.95 (0.88; 0.98)**	1.5	4.1
Pelvis—trunk (n = 25)	90.3 (3.3)	90.5 (3.4)	-0.2 (2.1)	-4.3; 3.9	0.81 (0.61; 0.91)**	1.5	4.1
Residual limb—trunk (n = 25)	179.1 (7.9)	178.6 (6.7)	0.4 (3.5)	-6.5; 7.3	0.89 (0.77; 0.95)**	2.4	6.7
***Healthy subjects***							
Pelvis—limb (n = 41)	83.0 (4.2)	83.0 (3.9)	0.0 (1.4)	-2.6; 2.8	0.95 (0.90; 0.97)**	1.0	2.6
Pelvis—trunk (n = 41)	90.3 (2.4)	89.7 (2.2)	0.6 (2.2)	-3.7; 4.9	0.54 (0.28; 0.72)**	1.9	5.4
Limb—trunk (n = 41)	172.7 (5.2)	173.3 (4.7)	-0.6 (2.6)	-5.7; 4.5	0.86 (0.75; 0.92)**	1.8	5.1

°: Degrees; SD: Standard deviation; Diff: Difference; LoA: limits of agreement; ICC: Intraclass correlation coefficient; CI: Confidence interval; SEM: Standard error of measurement; SDC: Smallest detectable change; %: percentage

*: significant at p < 0.05

**: significant at p<0.001. In 5 healthy subjects a technical error occurred which resulted in undetectable peak values due to an inconsistent waveform, hence resulting in 36 participants.

#### 2-D video-based system

**Fig 5:**
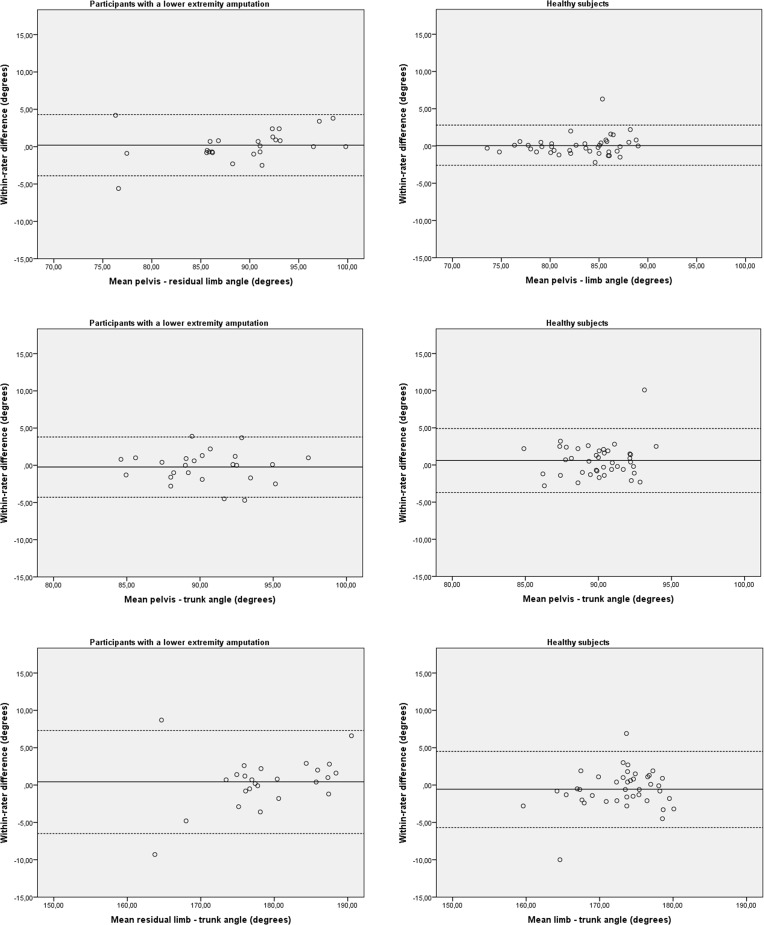
Bland–Altman plots for test-retest reproducibility of the 2-D video based system. The solid line represents the mean difference (systematic bias) and the dashed lines illustrate the 95% limits of agreement (mean difference ± 1.96 SD of the difference).

The test-retest reproducibility of the 2-D video-based system in group 1 and group 2 is detailed in Table 2. In group 1, the measured and calculated angles showed a very good to excellent reliability. The SEM and SDC ranged from 1.5° to 2.4° and 4.1° to 6.7°, respectively. In group 2, the angles showed a fair to excellent reliability. The SEM and SDC ranged from 1.0° to 1.9° and 2.6° to 5.4°, respectively. In both groups no bias in measurement error was detected as shown in the Bland-Altman plots (Fig 5).

### Inter-rater reproducibility

**Fig 6:**
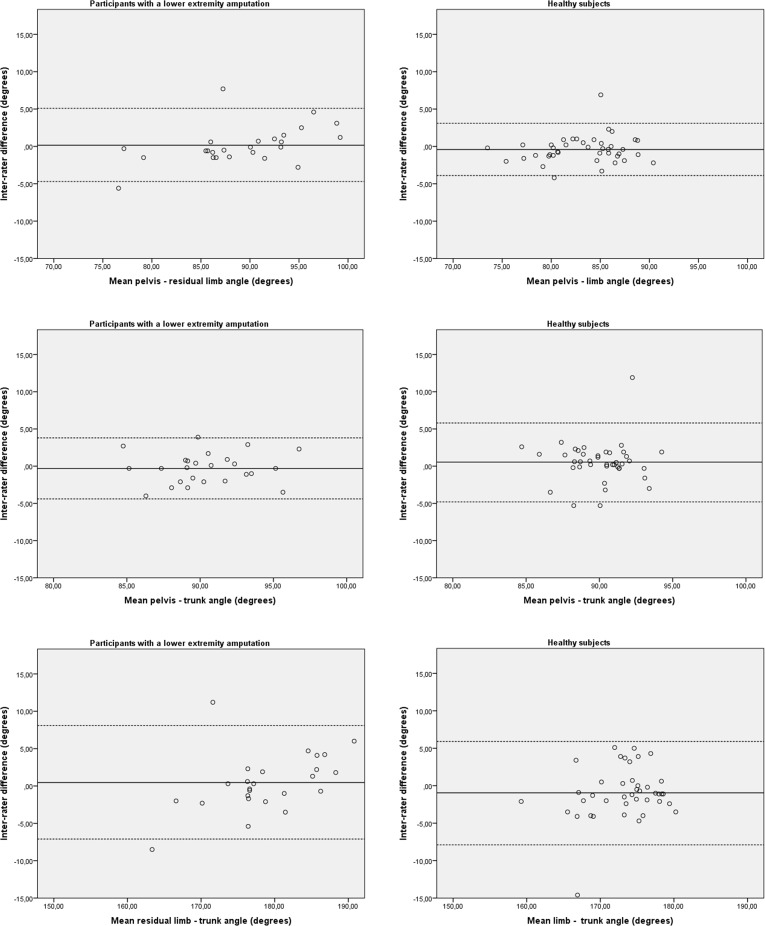
Bland–Altman plots for inter-rater reproducibility of the 2-D video based system. The solid line represents the mean difference (systematic bias) and the dashed lines illustrate the 95% limits of agreement (mean difference ± 1.96 SD of the difference).

The inter-rater reproducibility of the 2-D video-based system in group 1 and group 2 is detailed in Table 3. In group 1, the measured and calculated angles showed a good to very good reliability. The SEM and SDC ranged from 1.4° to 2.7° and 4.0° to 7.6°, respectively. In group 2, the angles showed a slight to very good reliability. The SEM and SDC ranged from 1.3° to 2.5° and 3.6° to 7.0°, respectively. In both groups no bias in measurement error was detected as shown in the Bland-Altman plots (Fig 6).

**Table 3:** Inter-rater reproducibility 2-D video-based system.

Angle	Tester 1 (°)	Tester 2 (°)	Diff test-retest (°)	95% LoA (°)	ICC2.1_agreement_ (95% CI)	SEM_agreement_	SDC _agreement_
	mean (SD)	mean (SD)	mean (SD)			(°)	(°)
***Participants with a lower extremity amputation***							
Pelvis—residual limb (n = 25)	89.3 (6.7)	89.2 (5.5)	0.2 (2.5)	-4.7; 5.1	0.92 (0.82; 0.96)**	1.8	4.9
Pelvis—trunk (n = 25)	90.3 (3.3)	90.6 (3.2)	-0.3 (2.1)	-4.4; 3.8	0.80 (0.60; 0.91)**	1.4	4.0
Residual limb—trunk (n = 25)	179.1 (7.9)	178.6 (6.2)	0.5 (3.9)	-7.1; 8.1	0.85 (0.69; 0.93)**	2.7	7.6
***Healthy subjects***							
Pelvis—limb (n = 41)	83.0 (4.2)	83.4 (3.9)	-0.4 (1.8)	-3.9; 3.1	0.90 (0.82; 0.95)**	1.3	3.6
Pelvis—trunk (n = 41)	90.3 (2.4)	89.8 (2.4)	0.5 (2.7)	-4.8; 5.8	0.39 (0.10; 0.62)*	1.9	5.4
Limb—trunk (n = 41)	172.7 (5.2)	173.6 (4.5)	-1.0 (3.5)	-7.9; 5.9	0.73 (0.55; 0.85)**	2.5	7.0

°: Degrees; SD: Standard deviation; Diff: Difference; LoA: limits of agreement; ICC: Intraclass correlation coefficient; CI: Confidence interval; SEM: Standard error of measurement; SDC: Smallest detectable change; %: percentage

*: significant at p < 0.05

**: significant at p<0.001

### Discriminant validity

#### IMU-system

In group 1, the within-group comparison (Table 4) revealed that the MARL was significant larger than the MASL with a mean difference of 0.5° (95% CI: 0.26; 0.80) in the low back IMU and 0.4° (95% CI: 0.12; 0.77) in the pelvic IMU. In group 2 the within-group comparison revealed no statistically significant difference between MAL and MAR in both IMU’s. The between-group comparison (Table 5) revealed that the MARL (group 1) was significant larger than the averaged maximum amplitude of group 2 with a mean difference of 1.2° (95% CI: 0.50; 1.89) in the low-back IMU. The MASL of the low back IMU, and the MARL and MASL of the pelvic IMU (group 1) showed no statistically significant differences compared to the averaged maximum amplitude of group 2. The mean differences between group 1 and 2 on these outcomes ranged from 0.1° to 0.7°.

**Table 4:** Within-group comparison IMU-system.

*Participants with a lower extremity amputation (n = 25)*				
IMU	Mean (SD) max amp to	Mean (SD) max amp to	Mean (SD)	95% Confidence
	residual limb side: MARL (°)	sound limb side: (MASL) (°)	difference (°)	Interval
Low back IMU	4.3 (1.3)	3.8 (1.2)	0.5 (0.7)	0.26; 0.80**
Pelvic IMU	4.7 (1.9)	4.3 (1.7)	0.4 (0.8)	0.12; 0.77*
***Healthy subjects (n = 36)***				
IMU	Mean (SD) max amp to left	Mean (SD) max amp to right	Mean (SD)	95% Confidence
	side: MAL (°)	side: MAR (°)	difference (°)	Interval
Low back IMU	3.1 (1.4)	3.2 (1.4)	-0.1 (0.7)	-0.36; 0.09
Pelvic IMU	5.5 (1.5)	5.3 (1.7)	0.2 (0.8)	-0.04; 0.52

Max amp: Maximum amplitude; %: percentage

*: significant at p < 0.05

**: significant at p < 0.001; Statistics based on the paired T-test

**Table 5:** Between-group comparison IMU-system.

	*Healthy subjects (n = 36)*	*Participants with a lower extremity amputation (n = 25)*					
IMU	Mean (SD) max amp	Mean (SD) max amp	Mean (SD) max amp	Mean difference	95%	Mean difference	95%
	of left and right (°)	to residual limb side:	to sound limb side:	MARL versus	Confidence	MASL versus	Confidence
		MARL (°)	MASL (°)	Healthy subjects	Interval	Healthy subjects	Interval
Lower back IMU^a^	3.1 (1.4)	4.3 (1.3)	3.8 (1.2)	1.2	0.50; 1.89*	0.7	0.00; 1.34
Pelvic IMU^b^	4.1 (1.1)	4.7 (1.9)	4.3 (1.7)	0.6	-0.28; 1.41	0.1	-0.66; 0.90

Max amp: Maximum amplitude; %: percentage

*: significant at p < 0.05

^a^Statistics based on the independent T-test

^b^Statistics based on the Welch test

#### 2-D video-based system

The between-group comparison (Table 6) revealed that group 1 had a larger pelvis—residual limb angle and residual limb—trunk angle than of group 2 with a mean difference of 6.3° (95% CI: 3.3; 9.3) and 6.4° (95% CI: 3.1; 9.6), respectively. The pelvis—trunk angle revealed no statistically significant difference between the two groups.

**Table 6:** Between-group comparison 2-D video-based system.

	*Group 2*	*Group 1*		
Angle	Healthy subjects (n = 41)	Participants with a lower	Mean difference	95%
	Mean (SD) angle (°)	extremity amputation (n = 25)	Group 2 vs	Confidence
		Mean (SD) angle (°)	Group 1	Interval
Pelvis—(residual) limb^b^	83.0 (4.2)	89.3 (6.7)	6.3	3.3; 9.3**
Pelvis–trunk^a^	90.3 (2.4)	90.3 (3.3)	-0.1	-1.5; 1.4
(Residual) limb–trunk^a^	172.7 (5.2)	179.1 (7.9)	6.4	3.1; 9.6**

%: percentage

**: significant at p < 0.001

^a^Statistics based on the independent T-test

^b^Statistics based on the Welch test

## Discussion

The IMU-system showed to be accurate in measuring coronal plane kinematics in both the low back and the pelvic IMU in an experimental setting. In a clinical setting, the IMU-system and 2-D video-based system showed to be reliable systems to measure the most coronal plane kinematic parameters during gait both in participants with a lower extremity amputation (group 1) as in healthy subjects (group 2). The asymmetry detected by the IMU-system in the low back and pelvic coronal plane kinematics in group 1 is in line with previous reported studies [7–12]. The between-group comparison revealed that only the MARL in the low back IMU and the (residual) limb—trunk angle were significantly larger than the maximum amplitude of healthy subjects. These results suggest that both systems are suitable for daily clinical practice with exception of the pelvic IMU, but the between-group comparison also revealed that the average difference was smaller than the SDC of the systems. The within-group asymmetry detected by the IMU system was also smaller than the SDC. So, it is questionable whether both systems will be sensitive enough to detect real differences within and between participants with a lower extremity amputation and healthy subjects.

The test-retest reproducibility of measuring coronal plane kinematics of the trunk is better compared to the pelvis if a 3-D motion capture system is used [56], which is similar as our findings with the IMU-system. The reproducibility parameters of our IMU-system are comparable as found in a 3-D motion capture system [56], however, likely the construct that is measured by the two systems differs. Previous studies which used a 3-D motion capture system reported a symmetry value of 2.1° for the trunk [9] and 1.9° to 2.0° for the pelvis [9,10] in individuals with a lower extremity amputation, which is much higher than the symmetry values found in our study. In addition, these higher symmetry values result in a more favourable ratio between the symmetry values and the SDC than with the IMU-system and the 2-D video-based system used in our study. The use of multiple surface markers per body segment versus one IMU as used in our study could explain the difference in measured construct. Another explanation for the relative low asymmetry values in our study could be that outcomes of IMU-systems are influenced by noise, limited resolution and constraints on mathematical calculations as concluded by Bauer et al.[41]. They used a similar IMU-system as we did to examine the validity of ROM tests of the trunk and concluded that the IMU-system underestimated the coronal plane movements ranging from 0.7° to 3.1°. So, our results may also be underestimated. The included patient population may also explain the relative low asymmetry values. We included both conventional socket-suspended prosthesis users as bone-anchored prosthesis users. Hypothetically, bone-anchored prosthesis users have less asymmetry in their gait than socket-suspended prosthesis users due to i.a. the fixed attachment of the prosthetic parts to the body. This was confirmed by Tranberg et al. [57] who assessed sagittal plane kinematic parameters. However, evidence about frontal plane kinematics is absent.

The construct we measured with our 2-D video-based systems is not measured before, which makes it difficult to put our results in perspective. Grunt et al. [58] assessed the reproducibility of sagittal plane measurements with a 2-D video-based system. The pelvic tilt angle was assessed compared to the laboratory floor and the trunk tilt angle was assessed compared to the pelvis. The pelvic tilt showed a similar test-retest reproducibility (ICC 0.92, SDC: 4.0°) but a worse inter-rater reproducibility (ICC 0.67, SDC: 8.5) than we found in measuring the pelvis—residual limb angle. The trunk tilt showed worse reproducibility for both the test-retest (ICC 0.77, SDC: 6.7°) and the inter-rater assessment (ICC 0.67, SDC: 8.5°) compared to our pelvis—trunk angle measurement.

### Strengths and limitations

A strength of this study is that we examined the reproducibility of an IMU system obtaining trunk and pelvic kinematic parameters during gait in a patient population. The reproducibility of IMU-systems is an underexposed topic and trunk and pelvic kinematic parameters are to our knowledge only examined in healthy subjects. Orlowski et al. [59] fixated an IMU on the pelvis and on the cervical spine during a gait measurement. Compared to our results they found lower and higher reliability for the trunk IMU (ICC: 0.76) and the pelvis IMU (ICC: 0.77), respectively. We estimated the SDC using their presented LoA and mean difference between the test and the retest, which is a slightly different method to calculate the SDC as we used. The SDC of the trunk IMU and the pelvic IMU were 1.7° and 2.2°, respectively. Our trunk IMU performed better and our pelvic IMU performed worse. A second strong point of our study is that we presented agreement parameters of the two examined systems, which enhances the interpretation by clinicians. Previous studies examining the reproducibility of IMU systems often presented reliability parameters (e.g. ICC’s) combined with a ‘coefficient of variation’ (CV) as a measure of agreement [41,60,61]. The CV is an inappropriate parameter to indicate the level of measurement error in the field of medicine, it is a measure to determine the level of reliability in the phase of calibration of an apparatus [52]. A Third strong point is that we examined simultaneous the discriminant validity and reproducibility of gait kinematics. Insight in both parameters raised questions about the clinical viability of both the IMU system and the 2-D video-based system, because the SDC was larger than the average difference between individuals with a lower extremity amputation and healthy subjects.

This study also contains limitations. First, we did not measure spatiotemporal parameters, therefore we can only interpret the symmetry values obtained with the IMU-system and not the MARL and MASL in relation to the phase of the gait cycle. This results in a limited usability for selecting the optimal therapy to increase the symmetry. Second, we did not use a fixed anatomical landmark for the low back IMU. We chose to standardise the measurement procedure using the applicator of the ValedoMotion with a fixed length of 17.5 cm. Due to differences in the height of the participants the low back IMU was positioned within a range of the thoracic spine which may have resulted in a larger variation in the obtained amplitudes. Moreover, it seems to be more difficult to interpret the construct of the gait adaptations used by participants with a lower extremity amputation. For instance, lateral bending of the trunk will lead to a larger range of motion than a lateral shift. Finally, we analysed the peak values of the filtered data instead of the waveform which is a more detailed analysis [32] and is commonly used to analyse IMU data [43,62–64]. We chose to use the peak values because this was possible for both the IMU-system and the 2-D video-based system. The peak value analysis also resulted in clinical feasible outcome measures such as the SDC, which is not the case for waveform similarity statistics.

Within the 2-D video-based analysis only one specific video frame was examined which is a poor representation of the entire gait cycle. Previous studies already concluded that a 2-D measurement measures a different construct than a 3-D measurement, probably due to additional movements in other planes [45,46]. Rotation deviations in the transversal plane of the limb, pelvis or trunk will influence the angles measured in the coronal plane. For example, an external rotation of the limb will lead to an overestimation of the pelvis—limb angle.

The criterion validity of trunk [49,65] and pelvic [43,49,62,64,65] IMU’s during various applications (e.g. gait) is established in healthy subjects, but it is necessary that this is also examined in individuals with a lower extremity amputation. Gait adaptations vary tremendously within persons, so it should be examined if the currently used two IMU’s and the chosen anatomical landmarks were optimal to identify gait adaptations. For example, expanding the number of IMU’s and the use of fixed anatomical landmarks may decrease the amount of measurement error. Longitudinal validity (responsiveness) of both the IMU-system and the 2-D video-based system should also be investigated before the systems are applicable for evaluating gait kinematics within a rehabilitation trajectory [66]. Most likely, a smaller average difference can be expected between subgroups of individuals with a lower extremity amputation and over time within rehabilitation trajectory than we found in our between–group comparison. Future research should establish this and the impact on the ratio between the symmetry values obtained with the IMU system and the (residual) limb—trunk angle obtained with the 2-D video-based system and their SDC.

## Conclusion

The IMU-system and 2-D video-based system tested in this study were not sensitive enough to detect real differences within and between participants with a lower extremity amputation and healthy subjects although promising reproducibility parameters for some of the outcome measures. It is therefore not likely that they will be suitable for evaluation of coronal plane kinematic parameters of the low back and pelvis in a clinical setting.

## Supporting information

S1 Text Appendix A. (PDF)

S1 File Data reproducibility and discriminant validity. (SAV)

S2 File Data accuracy ValedoMotion IMU-system. (SAV)
