# Supplementary material for: Reproducibility and discriminant validity of two clinically feasible measurement methods to obtain coronal plane gait kinematics in participants with a lower extremity amputation
Source: PLoS One. 2019 May 21;14(5):e0217046. doi: 10.1371/journal.pone.0217046 (PMC6528991; doi:10.1371/journal.pone.0217046)
Supplement: S1 Text — (PDF) [file pone.0217046.s001.pdf]

Appendix A

Methods

The aim of this experiment was to determine the accuracy of the IMU-system using a goniometer in an experimental setup (Fig A.1).

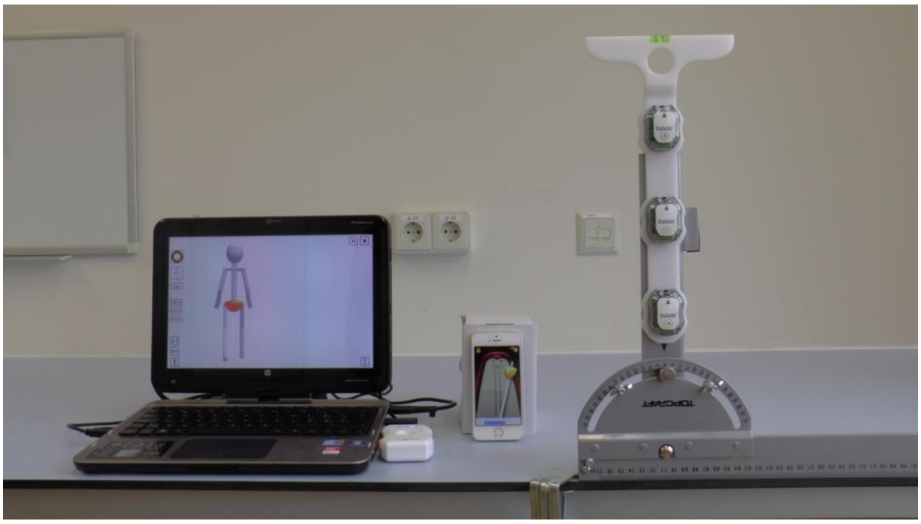

Fig A.1. Experimental setup

Left: notebook with receiver; Middle: metronome; Right: goniometer and reference holder with IMU's (Upper: reference IMU, Middle: low back IMU, Lower: pelvic IMU)

Study procedure

The accuracy experiment of the IMU-system (ValedoMotion, Hocoma, Volketswil, Switzerland) was performed by an experienced physiotherapist who was familiar with the IMU system (RL). All trials within the experiment were performed on the same day.

Testing procedure

The ValedoMotion IMU-system was developed for low back pain therapy but was modified by Hocoma for this research. As result of modification the IMU's raw data was exported to a comma-separated-value (csv)-file and we had access to a training application to provide patient's real-time visual feedback (Fig A.2). The IMU-system

consisted of three wireless IMU's containing a tri-axillar gyroscope, magnetometer, and accelerometer with a fixed axle system. Two of the IMU's were designed for positioning on an anatomical landmarks (low back and pelvis) using double-sided tape, the third IMU was designed for calibrating the low back and pelvic IMU using an additional reference holder including a level instrument (Fig A.3). In the calibration process the orientations of the IMU's following the magnetometer are used to determine the zero-angles, which are the measured rotation angles at which the patient is standing upright. The calibrated rotations were measured using a slightly variable sampling frequency around 300 Hz and exported to a notebook (Hewlett-Packard).

To examine the accuracy of the IMU system in the experimental setup, the three IMU's were placed in the reference holder which was fixed with double-sided tape onto a goniometer (Topcraft). The calibration process was performed while the goniometer was pointing upwards (Fig A.1). Within a trial ten consecutive fixed angle displacements in the coronal plane were made by the physiotherapist with a fixed cadence (1 Hz) guided by a metronome. The fixed angle displacements ranged from 1 degree to 30 degrees. At the start of each trial the IMU's were calibrated. Before the start of the experiment, the location of the experiment was checked for magnetic fields using the ValedoMotion software to prevent magnetic interferences influencing the measurements [1,2].

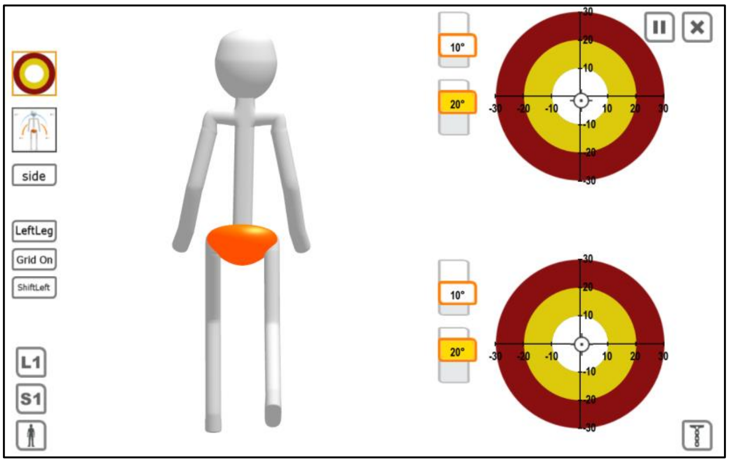

Fig A.2. Training application ValedoMotion

Left: avatar displaying the kinematics of the low back and pelvis IMU in real time; Right: adjustable bull's-eye displaying the limits of displacement of the low back (upper) and pelvic IMU (lower). An additional auditory cue can be provided if the patient shifts beyond the set limits.

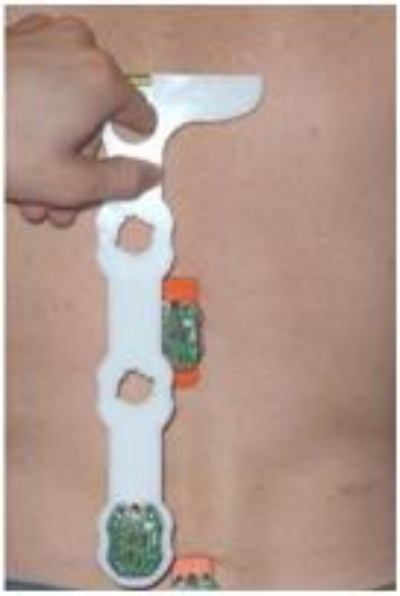

Fig A.3. Reference holder ValedoMotion

Data analysis

MATLAB (Release 2015b, The MathWorks Inc., Natick, Massachusetts, United States) was used (RL) to process the csv-file containing the raw data. Only the coronal plane kinematic parameters of the low back and pelvic IMU were analysed. A MATLAB protocol was used to automatically detect peak angles for each trial based on an expected cadence interval within 0.5 and 2 Hz; due to the nature of the automated detection, negative peak angles were identified in between two peak values. Therefore, each trial yielded five positive peak values (maximum amplitude to the left; MAL) and four negative peak values (maximum amplitude to the right: MAR) which could be processed (Fig A.4). Detected peaks were visually checked and corrected if necessary before proceeding. For each trial we calculated the average for both the MAL and the MAR. Additionally, the extent of asymmetry within each trial was calculated as the average difference between MAL and MAR, resulting in a symmetry value.

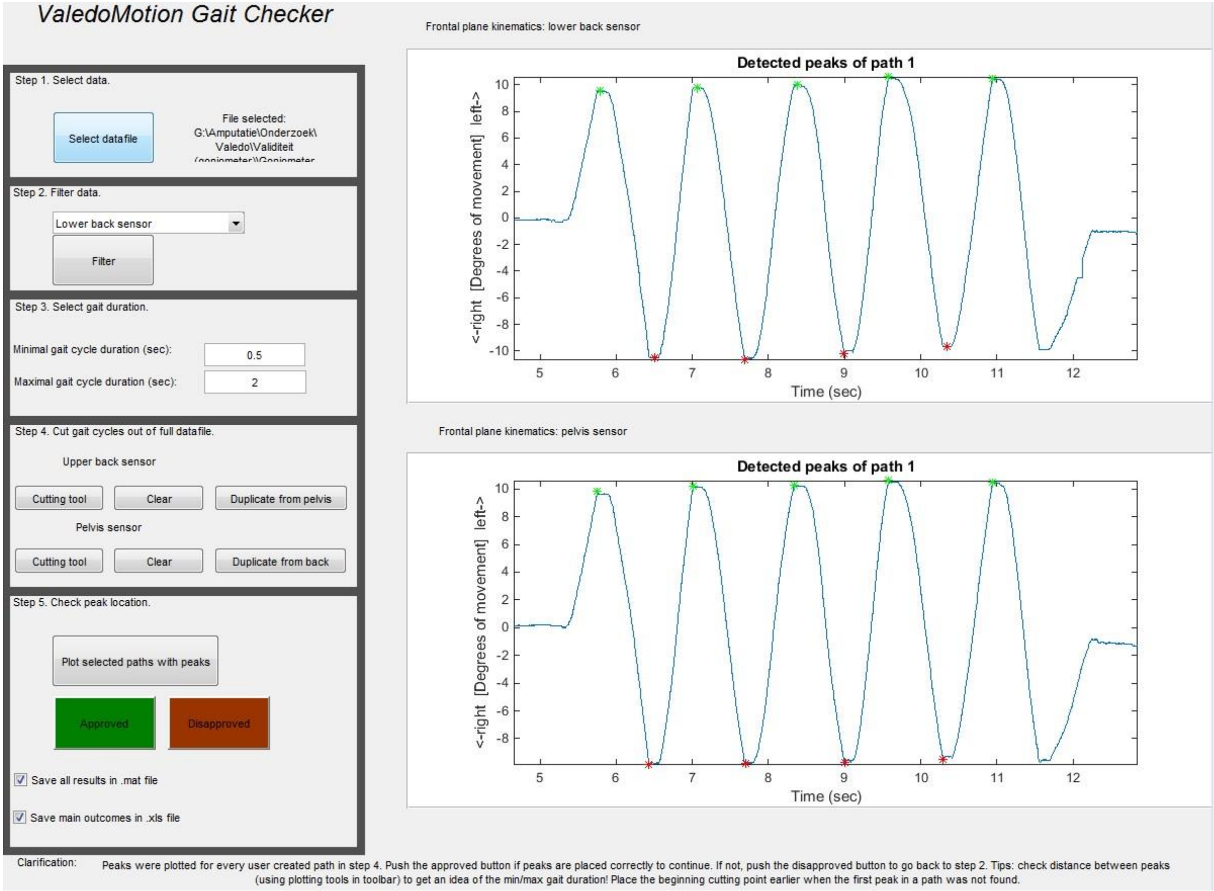

Fig A.4. Selection of the peak values using a MATLAB application

Example of the 10 degrees trial; Note that step 2 (filter data) was not used within the accuracy experiment to filter the angle output of the IMU's.

Statistical analysis

IBM SPSS Statistics v22 (SPSS Inc., Chicago, Illinois, United States) was used to calculate the group average including standard deviation for both the MAL, the MAR and the symmetry value using the trial averages, independently for the low back and pelvic IMU.

The difference in both the MAL and the MAR values (degrees) obtained with the IMU-system and the goniometer were calculated. The accuracy of both the low back and the pelvic IMU was determined by calculating the intraclass correlation coefficient (ICC) using a two-way mixed effects model ($ICC3.1_{consistency}$) with 95% confidence intervals (CI) [3]. The presence of bias in measurement error was assessed using a Bland-Altman plot including the 95% limits of agreement (95% LoA) [4,5]. The interpretation of the ICC values was based on guidelines offered by Byrt [6]: 0.01-0.20 poor accuracy, 0.21-0.51 slight accuracy, 0.41-0.60 fair accuracy, 0.61-0.80 good accuracy,

0.81-0.92 very good accuracy, and 0.93-1.00 excellent accuracy. In all cases, two sided p-values <0.05 were considered to be statistically significant.

Results

No technical errors occurred during data collection or data analysis resulting in no missing data in the 29 trials (1 degree to 30 degrees). Results of the comparison of the IMU-system and the goniometer (Table A.1 and A.2) revealed that the accuracy of the IMU system was excellent for both the low back and the pelvic IMU ($ICC3.1_{consistency}$: 1.00, 95% CI: 1.00-1.00). The 95% LoA was -1.08 to 0.59 degree and -0.58 to 0.98 degree for the low back IMU and the pelvic IMU, respectively. No bias in measurement error was detected in both IMU's (Fig A.5), but a slight asymmetry (0.2 to 0.7 degree) in maximum amplitude between left and right was present as shown in Table A.1 and A.2.

Table A.1. Accuracy maximum amplitude low back IMU

Type of device	Max Amp (°) mean (SD)	Diff GM-VM (°) mean (SD)	95% LoA (°)	$ICC3.1_{consistency}$ (95% CI)
	**Movement to left**			
ValedoMotion (n = 30)	15.5 (8.7)	0.0 (0.3)	-0.59; 0.59	1.00 (1.00; 1.00)**
Goniometer (n = 30)	15.5 (8.8)			
	**Movement to right**			
ValedoMotion (n = 30)	15.8 (9.0)	-0.4 (0.6)	-1.08; 0.48	1.00 (1.00; 1.00)**
Goniometer (n = 30)	15.5 (8.8)			
	**Symmetry (left - right)**			
ValedoMotion (n = 30)	-0.7 (1.2)	NA	NA	NA
Goniometer (n = 30)	0 (0)			

Max Amp: Maximum amplitude; Diff: Difference; VM: ValedoMotion; GM: Goniometer; LoA: Limits of agreement; ICC: Intraclass correlation coefficient; CI: Confidence; NA: Not applicable; °: degree; **: p<0.001

Table A.2. Accuracy maximum amplitude pelvic IMU

Type of device	Max Amp (°) mean (SD)	Diff GM-VM (°) mean (SD)	95% LoA (°)	$ICC3.1_{consistency}$ (95% CI)
	**Movement to left**			
ValedoMotion (n = 30)	15.3 (8.5)	0.2 (0.4)	-0.58; 0.98	1.00 (1.00; 1.00)**
Goniometer (n = 30)	15.5 (8.8)			
	**Movement to right**			
ValedoMotion (n = 30)	15.2 (8.7)	0.3 (0.3)	-0.29; 0.90	1.00 (1.00; 1.00)**
Goniometer (n = 30)	15.5 (8.8)			
	**Symmetry (left - right)**			
ValedoMotion (n = 30)	0.2 (1.5)	NA	NA	NA
Goniometer (n = 30)	0 (0)			

Max Amp: Maximum amplitude; Diff: Difference; VM: ValedoMotion; GM: Goniometer; LoA: Limits of agreement; ICC: Intraclass correlation coefficient; CI: Confidence; NA: Not applicable; °: degree; **: p<0.001

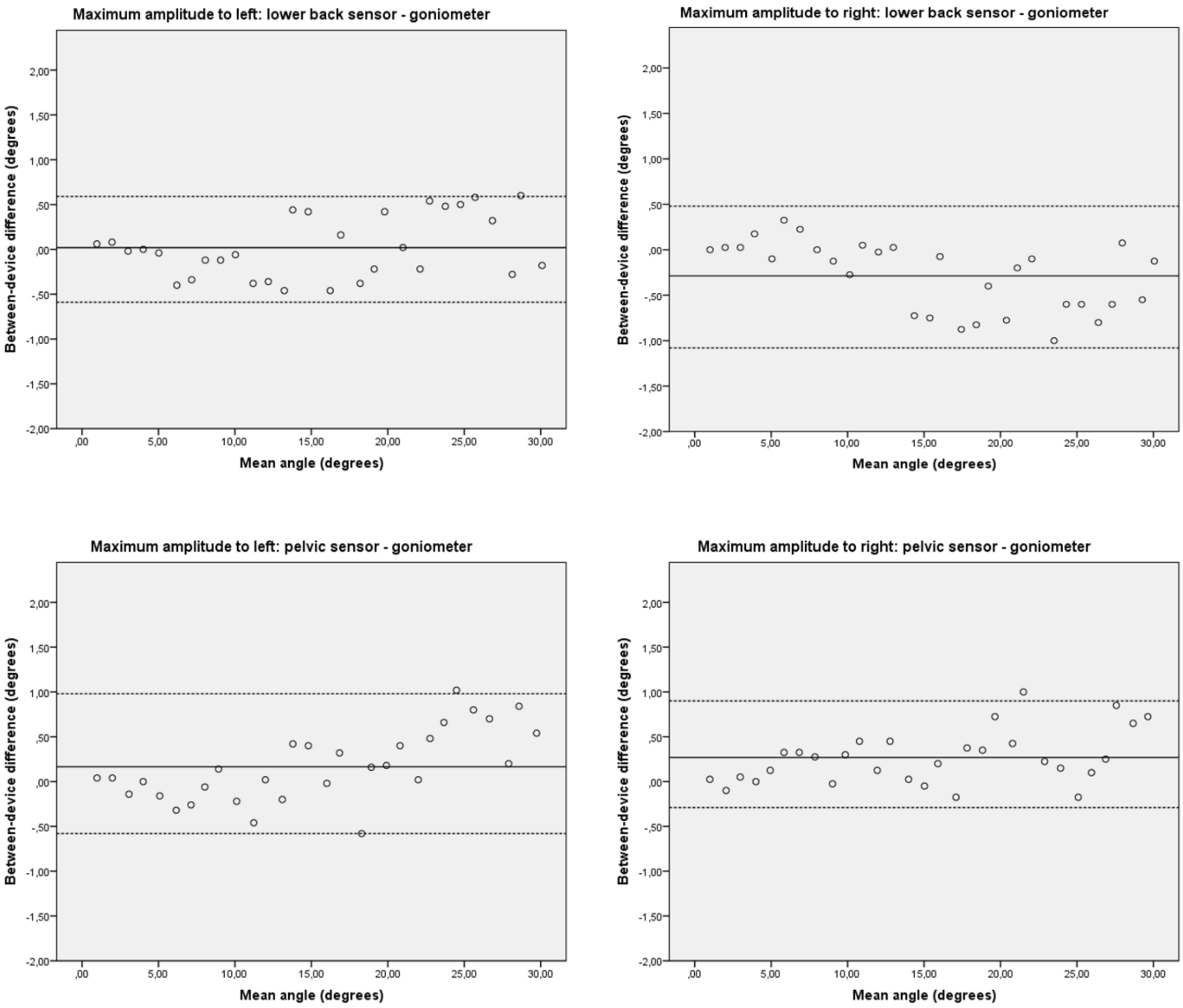

Fig A.5. Bland–Altman plots for between-device differences and their relation to the magnitude of the maximum amplitude measured with the IMU-system and the goniometer.

The solid line represents the mean difference (systematic bias) and the dashed lines illustrate the 95% limits of agreement (mean difference ± 1.96 SD of the difference)

References

1. Roetenberg D, Baten CT, Veltink PH. Estimating body segment orientation by applying inertial and magnetic sensing near ferromagnetic materials. IEEE Trans Neural Syst Rehabil Eng. 2007; 15(3): 469-71.

2. de Vries WH, Veeger HE, Baten CT, van der Helm FC. Magnetic distortion in motion labs, implications for validating inertial magnetic sensors. Gait & posture. 2009; 29(4): 535-41.

3. Atsma F, Bartelink ML, Grobbee DE, van der Schouw YT. Best reproducibility of the ankle-arm index was calculated using Doppler and dividing highest ankle pressure by highest arm pressure. J Clin Epidemiol. 2005; 58(12): 1282-8.

4. Portney LG, Watkins MP. Foundations of Clinical Research - Applications to Practice. New Jersey: U.S.A.: Pearson Education; 2009.

5. Bland JM, Altman DG. Measuring agreement in method comparison studies. Stat Methods Med Res. 1999; 8(2): 135-60.

6. Byrt T. How good is that agreement? Epidemiology. 1996; 7(5): 561.
